# Prevalence of neonatal near miss in Africa: a systematic review and meta-analysis

**DOI:** 10.1093/inthealth/ihad034

**Published:** 2023-05-10

**Authors:** Teklehaimanot Gereziher Haile, Dawit Gebregziabher, Gebreamlak Gebremedhn Gebremeskel, Guesh Mebrahtom, Woldu Aberhe, Abrha Hailay, Kidane Zereabruk, Gebremeskel Tukue Gebrewahd, Tamirat Getachew

**Affiliations:** S chool of Nursing, College of Health Sciences, Axum University, Tigray, Ethiopia; Department of Maternity and Reproductive Nursing, School of Nursing, Aksum University, Aksum, Ethiopia; Department of Adult Health Nursing, School of Nursing, Aksum University, Aksum, Ethiopia; Department of Adult Health Nursing, School of Nursing, Aksum University, Aksum, Ethiopia; Department of Adult Health Nursing, School of Nursing, Aksum University, Aksum, Ethiopia; Department of Adult Health Nursing, School of Nursing, Aksum University, Aksum, Ethiopia; Department of Adult Health Nursing, School of Nursing, Aksum University, Aksum, Ethiopia; Department of Emergency Medicine and Critical Care Nursing, School of Nursing, Aksum University, Aksum, Ethiopia; School of Nursing and Midwifery, College of Health and Medical Sciences, Haramaya University, Harar, Ethiopia

**Keywords:** Africa, near miss, neonatal, newborn, prevalence

## Abstract

**Background:**

Neonatal near miss (NNM) applies to cases where newborns almost died during the first 28 d of life but survived life-threatening conditions following birth. The most vulnerable time for infant survival is the neonatal stage, corresponding to almost 50% of deaths occurring at <5 y of age. No study indicates the overall pooled prevalence of NNM in Africa. Thus this review aimed to estimate the overall pooled prevalence of NNMs in Africa.

**Methods:**

Articles were retrieved through a comprehensive search strategy using PubMed/MEDLINE, Embase, Health InterNetwork Access to Research Initiative, Cochrane Library and Google Search. Data extraction was done independently by all authors. Forest plots and tables were used to represent the original data. The statistical heterogeneity was evaluated using I^2^ statistics. There was heterogeneity between the included articles. Therefore the authors used a meta-analysis of random effects to estimate the aggregate pooled prevalence of NNM in Africa. Funnel plot and Egger regression test methods were used to assess possible publication bias. R software version 3.5.3 and R studio version 1.2.5003 were used to analyse the data. The guideline of the Preferred Reporting Items for Systematic Reviews and Meta-Analyses was used to publish this article. The review was registered on the International Prospective Register of Systematic Reviews (registration ID: CRD42021290223).

**Results:**

Through an exhaustive search, we found 835 articles. However, we considered only eight full-text articles to be included in this meta-analysis. The analysis of included studies showed that the overall pooled prevalence of NNM in Africa was 30% (95% confidence interval [CI] 16 to 44). The subgroup analysis by study year showed that the prevalence of NNM from 2012–2015 and 2018–2019 was 36% (95% CI 23 to 49) and 20% (95% CI 1 to 39), respectively.

**Conclusion:**

This finding suggests that the pooled prevalence of NNM is high in Africa as compared with other studies. Therefore the government and other stakeholders working on maternal and child health should assist in the design of interventions and strategies for improving the quality of neonatal care.

## Introduction

A neonatal near miss (NNM) applies to cases where newborns almost died during the first 28 d of life but survived life-threatening conditions following birth.^1–3^ The idea of near miss has been used in the field of maternal health as a tool for evaluating and improving the standard of treatment. In contrast, newborns with severity markers at birth but who survive NNM are known as neonatal age survivals.^1,4^ The most vulnerable time for infant survival is the neonatal stage, corresponding to almost 50% of deaths occurring at <5 y of age.^5^

More recently, the World Health Organization (WHO) has established a series of standard NNM markers to promote perinatal care quality assessments.^2,6^ Complications resulting from preterm birth, asphyxia during childbirth and sepsis, corresponding to 75% of these deaths, are the major causes of death in the neonatal period worldwide.^6^ It is understood that most neonatal deaths are preventable, and investing in maternal and neonatal care during gestation and in the first 24 h after birth is the most effective way to minimize these deaths.^7^ Globally, despite a remarkable achievement in mortality reduction in children <5 y of age, the proportion of neonatal mortality has increased from 41% to 46% from 2000 to 2016.^8^

While the Millennium Development Target for child survival was developed to reduce maternal and neonatal morbidity and mortality to <30 per 1000 live births by 2015, the global mortality rate of 41% in children <5 y of age decreased until 2011.^9^ The 2015 global report found that of the 2.7 million deaths of children <5 y of age, approximately 1 million deaths occurred during the first week of the neonatal period.^9,10^ In terms of reducing child mortality, remarkable progress has been made around the world. However, child survival remains an urgent concern. Globally, in 2017, 5.4 million children died before their fifth birthday and about half (47%) of these deaths occurred in the first month of life.^11–13^

The United Nations Agenda for Sustainable Development aimed to end preventable deaths of newborns by 2030 and suggested that neonatal mortality should be <12 per 1000 live births by the end of 2030. The largest number of neonatal deaths occurred in South-Central Asian and sub-Saharan African countries.^14,15^ The neonatal morbidity rate remains elevated despite a decrease in the neonatal mortality rate, especially in low- and middle-income countries. Some reports indicate that the number of newborn babies surviving an NNM event has been approximately three to six times higher than the number of newborn babies who have already died.^3,16–18^

The prevalence of NNM varies widely across studies because of the difference in criteria used to define the NNM.^19^ In studies that used only pragmatic criteria, the incidence of NNM ranged from 21.4 to 86.7 per 1000 live births in Brazil and India, respectively.^16,20^ But according to studies that used pragmatic and management criteria, the incidence of NNM ranged from 39.2 to 367 per 1000 live births.^21,22^

Although there have been various single studies conducted on the prevalence of NNM in different countries of Africa, they present inconsistent findings and there has been no strong study done to indicate the overall pooled prevalence of NNM in Africa. Thus this review aimed to estimate the overall pooled prevalence of NNM in Africa.

## Methods

### Protocol and registration

This review was based on the Preferred Reporting Items for Systematic Reviews and Meta-Analyses (PRISMA) guideline^23^ (Supplementary file 1). The review was registered in the International Prospective Register of Systematic Reviews (registration ID: CRD42021290223).

### Data source and search strategy

Articles were retrieved through a comprehensive search strategy using PubMed/MEDLINE, Embase, Health InterNetwork Access to Research Initiative (HINARI) and Cochrane Library databases. In addition, non-electronic sources were used, combined with Google Search, Google Scholar and MedNar to retrieve further articles. Searches were performed using each database on 10–12 November 2020. The quest was performed either individually or in combination using the following keywords: neonatal, prevalence, infant, newborn and each country's name ((‘infant, newborn’[MeSH Terms] OR (‘infant’[All Fields] AND ‘newborn’[All Fields]) OR ‘newborn infant’[All Fields] OR ‘neonatal’[All Fields]) AND (near [All Fields] AND miss [All Fields])) AND (‘Africa’[MeSH Terms] OR ‘Africa’[All Fields]). The search strategies for the Cumulative Index of Nursing and Allied Health Literature, HINARI and Google Scholar are outlined in Supplementary file 2. The search was also conducted by combining the above search terms in each country in Africa. All identified keywords and index terms were then checked across all databases. Finally, the reference lists of all identified articles were searched for further articles.

### Data extraction and quality assessment

The nine authors extracted the data independently using a structured method of data collection and a pre-piloted data extraction format prepared in an Excel spreadsheet (Microsoft, Redmond, WA, USA). The titles, abstracts of all citations obtained and full-text search results to classify potentially qualifying studies were independently reviewed by two reviewers (TGH and GGG). Data extraction included title, author's name, year published, research type, research base (population or hospital based), criteria (definitions) used for the studies, sample size, prevalence, response rate and study area. The quantitative data (the total sample size [N] and frequency of the event [n]) and effect size were extracted from the included articles and summarized using Excel 2016 for meta-analysis and synthesis. The Newcastle–Ottawa Quality Assessment Scale was used to assess the quality of all studies.^24^ This scale is primarily formulated by a star allocation system, assigning a maximum of 10 stars for the risk of bias in three areas: selection of study groups (4 or 5 stars), comparability of groups (2 stars) and ascertainment of the outcome of interest or the exposure (3 stars). The combined quality score established that 0–3, 4–6 and 7–10 stars would be considered as high, moderate and low risk of bias, respectively. Disagreements between the two reviewers were settled through dialogue and discussion.

Although different studies used different criteria, NNM was defined with the following criteria:

- Pragmatic: defined as the combination of variables including birthweight <1.7 kg, APGAR score <7 and gestational age <33 weeks.^2^
- Management: defined as the use of parenteral antibiotic treatment, nasal continuous supportive airway pressure (NCPAP), any intubation during the first week of life, phototherapy within 24 h of birth, cardiopulmonary resuscitation, vasoactive drug use, anticonvulsants use, surfactant use, blood product use, steroid use and surgery during the first week of birth.^25^
- Organ-based dysfunction and clinical severity: included severe anaemia; uterine rupture or Bandl's ring; severe haemorrhage; severe infection, jaundice or cardiac arrest; and severe pre-eclampsia and eclampsia.

### Inclusion and exclusion criteria

Inclusion criteria were

all published observational studies

 conducted in Africa,

in the English language,

published through 16 October 2020 and

 reporting the prevalence of NNM. Studies that did not meet the criteria were excluded.

### Publication bias and heterogeneity

The statistical heterogeneity was evaluated using I^2^ statistics. Heterogeneity was classified with values of I^2^ of 25%, 50%, and 75%, being representative of low, medium and high heterogeneity, respectively.^26^ There was heterogeneity between the included articles, thus the authors used a meta-analysis of random effects to estimate the aggregate pooled prevalence of NNM in Africa. Funnel plot and Egger regression test methods were used to assess possible publication bias. A p-value <0.05 indicated the presence of significant publication bias.^27^

### Critical appraisal of studies

The methodology and quality of the findings of the included studies were critically evaluated using the Quality Assessment Tool for Observational Studies (cross-sectional, case–control and cohort studies) developed by the Joanna Briggs Institute (JBI)^28^ (Supplementary file 3). Two author groups (TG and TGH, and GGG and DG) independently evaluated the quality of the studies. The mean score of the two groups was taken for a final decision. Differences in the inclusion of studies were resolved by consensus. The included studies were evaluated against each indicator of the tool and categorized as high (>80%), moderate (60–80%) or low quality (<60%). Studies with a score ≥60% were included. This critical appraisal was conducted to assess the internal validity (systematic error) and external validity (generalizability) of studies and to reduce the risk of bias.

### Statistical analysis and presentation of results

R version 3.5.3 and RStudio version 1.2.5003 )R Foundation for Statistical Computing, Vienna, Austria) were used to analyse the data. Relevant data from every study extracted in the Excel spreadsheet were used to estimate the pooled prevalence of NNM and the degree of statistical heterogeneity between articles. A funnel plot and Egger's test were used to assess publication bias. The pooled estimate was computed using the ‘meta prop’ command. I^2^ test statistics were used to assess the percentage of variations between studies’ results from heterogeneity. Results were presented using tables and forest plots with 95% confidence intervals (CIs). A random effects model was used due to the high level of heterogeneity among included articles.

### Data management

A framework was developed a priori to guide the screening and selection process, based on the criteria for inclusion and exclusion. The tool was piloted and revised before data extraction began. To delete duplicates, the search results were uploaded to EndNote software (Clarivate, Philadelphia, PA, USA).

### Data items

Data extraction included the first author, year published, country, sample size, type of publication, study area, criteria (definitions) used for the studies, prevalence and characteristics of the study (study design, response rate).

The primary outcome was the prevalence of NNMs in Africa.

### Data synthesis

The original articles were represented using a forest plot and table. Because there was heterogeneity among the studies, a random effects model^29^ was used to determine the pooled prevalence of NNM in Africa. Subgroup analyses were also conducted by different study characteristics, such as study country and year of publication. Heterogeneity was assessed using Cochrane's Q and quantified by I^2^ statistics.^26^ Results with corresponding 95% CIs were presented as proportions. The findings of this analysis were reported based on the PRISMA guideline.^23^

## Results

### Screening flow

**Figure 1.**
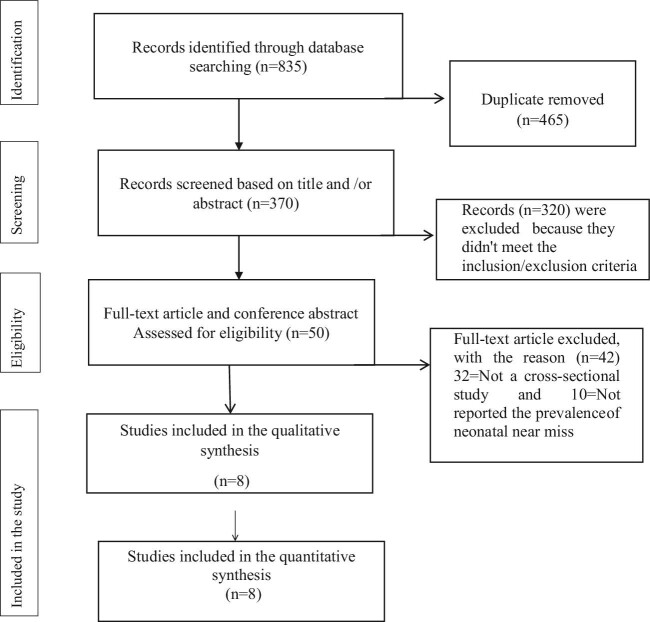
Selection of studies for a systemic review and meta-analysis of the prevalence of NNM in Africa.

Through an exhaustive search and discovery, we found 835 articles and removed 465 duplicates. Subsequently we reviewed 370 articles based on titles and abstracts and 320 articles were excluded because the studies did not meet the inclusion criteria (302 based on their titles and 18 based on abstracts). Fifty articles were assessed further and 42 articles were excluded, as 32 of the studies were not observational studies and 10 did not report the prevalence of NNM. Finally, based on the predefined criteria and quality assessment, we considered eight full-text articles with 7798 total samples of NNM, which were included in this systematic review and meta-analysis. The detailed steps of the screening process are shown in a PRISMA flow chart of study selection (Figure 1).

### Study characteristics

In this meta-analysis, a total of eight studies were included with a total sample size of 7798; the smallest sample size (378) was reported from a study conducted in Morocco^30^ and the largest sample size (2704) was reported from a study conducted in Ethiopia.^31^ A total of 37.5% of the studies were conducted in Ethiopia. Except for two studies (cross-sectional), all of the studies were prospective cohorts. The highest prevalence of NNM was found in Ghana (69.5%)^32^ and the lowest prevalence was in Ethiopia (4.51%).^31^ The included studies mainly used pragmatic, clinical and management criteria to diagnose NNM. The quality score of each primary study, based on the Newcastle–Ottawa Scale, showed no considerable risk, therefore all the studies were considered in this systematic review and meta-analysis. The detailed characteristics of included articles are presented in Table 1.

**Table 1.** Characteristics of studies included in the systematic review and meta-analysis of the prevalence of NNM in Africa, 2020

Author	Study year	Study area/country	Study design	Criteria (definitions) used for the studies	Sample size	Cases	Prevalence (%)	Quality score
Tekelab et al.^31^	July–November 2018	Ethiopia	Prospective cohort	Pragmatic criteria, clinical criteria and management criteria (APGAR <7, GA <33 weeks, birthweight <1750 g, cyanosis, RR >70 bpm, irregular breathing, cardiac arrest, bradycardia, jaundice, tachycardia, seizures, non-traumatic bleeding, haematuria, anuria >24 h, apathy, abdominal distension and vomiting, any intubation, CPR, use of vasoactive drugs, blood transfusion, use of anticonvulsant, phototherapy, parenteral antibiotics)	2704	122	4.51	Medium
Gebrehana Belay H. et al.^26^	February–April 2019	Ethiopia	Cross-sectional	Management criteria and pragmatic criteria (birthweight <2500 g, GA <37 weeks; 5-min APGAR score <7; use of mechanical ventilation, CPR, intubation, NCPAP, parenteral antibiotics, parenteral nutrition, vasoactive drugs, phototherapy, anticonvulsants, blood products, hypoglycaemia, surgical procedures and antenatal steroids)	404	94	23.3	Medium
Habtamu Abie T. et al.^27^	July 2018–June 2019	Ethiopia	Cross-sectional	Management criteria and pragmatic criteria (birthweight <1750 g, GA <33 weeks, 5-min APGAR score <7, newborn resuscitated with bag and mask, CPR, NCPAP, use of parenteral antibiotics, parenteral nutrition, vasoactive drugs, phototherapy, anticonvulsants and blood products or steroids)	422	139	32.9	High
C. Ronsmans et al.^30^	March–September 2012	Benin	Prospective cohort	Organ-based dysfunction and clinical severity criteria (severe anaemia; uterine rupture or Bandl's ring; severe haemorrhage; severe infection, jaundice or cardiac arrest; severe pre-eclampsia or eclampsia)	668	181	27.10	High
C. Ronsmans et al.^30^	May–November 2012	Burkina Faso	Prospective cohort	Organ-based dysfunction and clinical severity criteria (severe anaemia; uterine rupture or Bandl's ring; severe haemorrhage; severe infection, jaundice or cardiac arrest; severe pre-eclampsia or eclampsia)	686	131	19.10	High
C. Ronsmans et al.^30^	February 2012–January 2013	Morocco	Prospective cohort	Organ-based dysfunction and clinical severity criteria (severe anaemia; RR >70; uterine rupture or Bandl's ring; severe haemorrhage; severe infection, jaundice or cardiac arrest; severe pre-eclampsia or eclampsia)	378	115	30.4	Medium
Bakari et al.^24^	April—July 2015	Ghana	Prospective cohort	Management markers, pragmatic category and Organ-based dysfunction criteria	394	274	69.5	High
Nakimuli et al.^1^	1 March 2013 and 28 February 2014	Uganda	Prospective cohort	Clinical and pragmatic criteria (APGAR score ≤7, GA<30, birthweight <1500 g, use of mechanical ventilation; admission to NICU; blood transfusion; phototherapy; use of NCPAP; intubation; CPR; phototherapy; use of surfactant, parenteral antibiotic administration in the first 48 h of life; neonatal convulsions, neonatal respiratory morbidity; hypoglycaemia or necrotizing enterocolitis)	2142	730	34.1	High

CPR: cardiopulmonary resuscitation; GA: gestational age; NICU: neonatal intensive unit; RR: respiratory rate.

### The pooled prevalence of NNM in Africa

**Figure 2.**
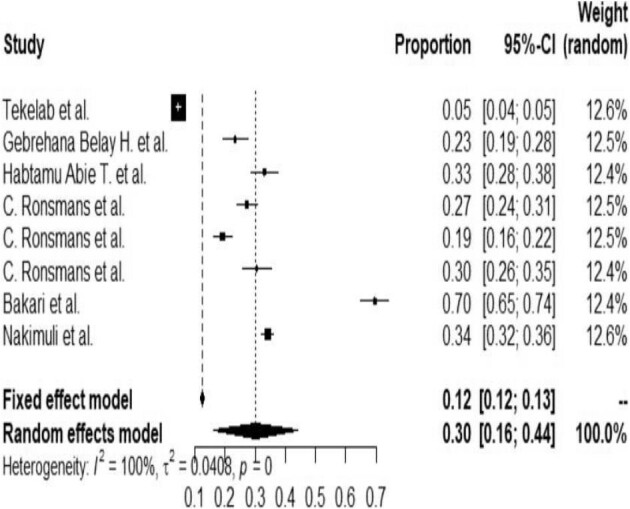
Forest plot that assessed the pooled prevalence of NNM in Africa, 2020.

The analysis of eight studies according to the random effects model showed that the overall pooled prevalence of NNM in Africa was 30% (95% CI 16 to 44). In this study, the heterogeneity was tested and I^2^ was 100%, indicating the presence of high heterogeneity (Figure 2).

### Subgroup analysis by country and study year

**Figure 3.**
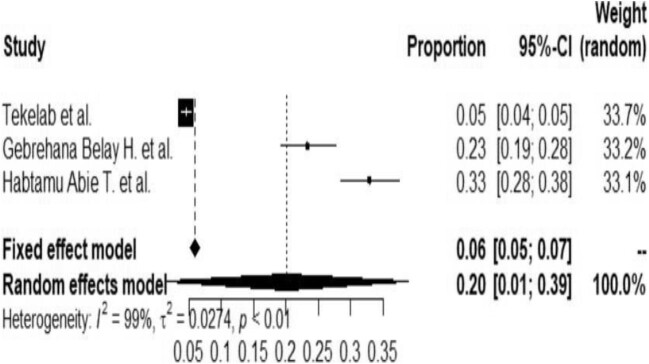
Forest plot for the subgroup analysis of Ethiopia.

Subgroup analysis was done by study country and year of publication with the evidence of marked heterogeneity to minimize the potential random variations between the studies included. Thus most studies were carried out in Ethiopia. The pooled prevalence of NNM in the subgroup analysis in Ethiopia was 20% (95% CI 1 to 39) (Figure 3).

**Figure 4.**
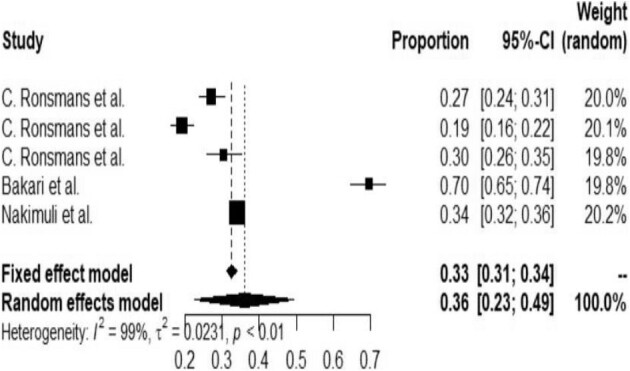
Forest plot for the subgroup analysis of NNM in Africa from 2012 to 2015.

**Figure 5.**
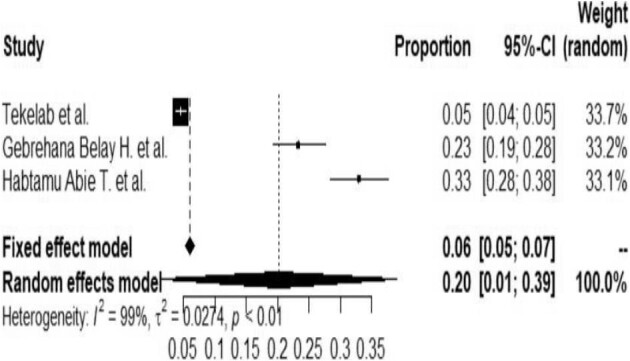
Forest plot for the subgroup analysis of NNM in Africa from 2018 to 2019.

Despite a very limited number of studies, a subgroup analysis was conducted based on the year of the studies. This showed a decrease in the prevalence of NNM from 36% (95% CI 23 to 49) (Figure 4) in 2012–2015 to 20% (95% CI 1 to 39) in 2018–2019 (Figure 5).

### Publication bias

**Figure 6.**
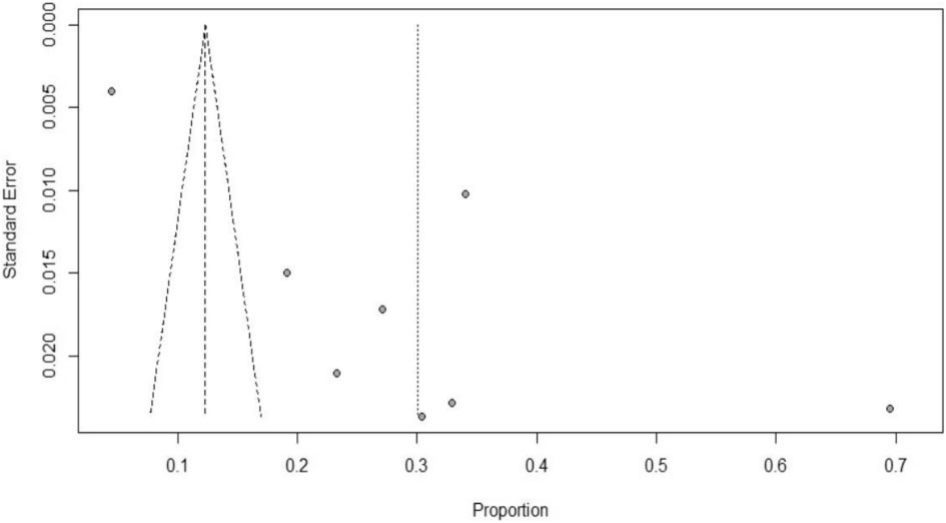
Funnel plot indicating publication bias.

Publication bias was assessed using a funnel test. The funnel plot in Figure 6 indicates the presence of publication bias. This indicated that there are unpublished articles that could modify the prevalence of NNM. Egger's test was also performed.

### Meta-regression to check the heterogeneity

A meta-regression analysis was conducted, as there was statistically significant heterogeneity, with I^2^ <0.05. The purpose of the analysis was to identify the source of heterogeneity so that the correct interpretation of the findings is made. However, the meta-regression analysis found no significant variable that can explain the heterogeneity. This includes the sample size, publication year and setting of included studies. Therefore the heterogeneity may be explained by other factors that these studies did not capture (Table 2).

**Table 2.** Meta-regression analysis to check heterogeneity of NNM in Africa, 2020

Variables	Coefficients	Standard error	p-Value	95% CI
Publication year	0.002466	0.0103598	0.817	−0.0206168 to 0.0255492
Sample size	−0.0000138	8.67e-06	0.144	−0.0000331 to 5.56e-06
Setting	0.1033236	0.0993437	0.323	−0.118028 to 0.3246752
Country	0.039861	0.0312109	0.230	−0.0296812 to 0.1094032

## Discussion

This systematic review and meta-analysis were performed to determine the overall pooled prevalence of NNM in Africa. Based on the findings of this meta-analysis, the pooled prevalence of NNM in Africa was estimated at 30% (95% CI 16 to 44). This finding has great implications for surviving and thriving neonates, combating preventable disease and future development and productivity, especially in developing countries. Individually, the prevalence of NNM in publications included in this study ranged from 4.51% reported by Tekelab et al.^31^ to 69.5% reported by Bakari et al.^32^ The explanation for such differences may be due to the quality of services and evaluation methods. This analysis highlights the major and urgent need to concentrate on the epidemiology of NNM in Africa to fully understand the situation and undertake relevant action plans that will reduce mortality. Moreover, this might be due to the use of less sensitive criteria for NNM, as the association between pragmatic and management criteria most probably permitted the evaluation of a larger number of surviving newborns considered to be at risk. It is important to highlight that NNM criteria are unable to identify the total number of neonatal deaths, using either pragmatic, management or a combination of these criteria at birth.

The pooled prevalence of NNM in this study is much higher than a comparative cross-sectional study conducted in Brazil, which found that the prevalence of NNM was 304 (14.5%) and 243 (9.9%) in the years 2012 and 2016, respectively.^33^ Similarly, studies conducted in northeast Brazil,^34^ the WHO multicounty survey^35^ and the Brazil survey^16^ indicated that the prevalence of NNM was 8.65%, 7.25% and 3.3%, respectively, which were lower than in the current study. The explanation for the difference might be due to variation in the NNM definition, differences in culture and economic status, differences in lifestyle and the general public's level of education. On the other hand, our overall pooled prevalence (30%) slightly coincides with a study conducted in northeastern Brazil (22%).^22^ This was probably due to the higher proportion of low-income countries contributing to the sample, resulting in worse neonatal conditions.

After looking at the heterogeneity of studies included in this systematic review and meta-analysis, a subgroup analysis was conducted based on the year of the included studies, which showed a decrease in the prevalence of NNM from 36% in 2012–2015 to 20% in 2018–2019. The difference might be related to the time that has passed since the studies were undertaken, as the government has implemented strategies such as introducing newborn care practices,^5^ increasing the number of health professionals^5^ and expanding neonatal intensive care units^36^ in recent years.

The implication of this study, particularly the pronounced variation between studies (4.51% to 69.5%), is to develop standard guidelines for the management of NNMs in clinical practice. In addition, and to the best of our knowledge, this is the first systematic review and meta-analysis providing a clear image of the pooled prevalence of NNM in Africa.

### Limitations of this study

It is very difficult to conduct a subgroup analysis of studies among countries because of the limited number of studies. Additionally, the case definition criteria were different from one study to another, thus the methodological variations between the studies included in this review could also affect the results of the meta-analysis, with far-reaching clinical heterogeneity across studies.

### Conclusions

This study systematically summarizes the pooled prevalence of NNM in Africa. This finding suggests that the pooled prevalence of NNM in Africa is high as compared with previous studies. Each country's ministry of health, health policymakers, clinicians and other healthcare providers should focus on strengthening the quality of healthcare services for neonates to reduce mortality. The authors believe that this systematic review and meta-analysis provides concrete evidence at a national level in Africa and will assist in the design of interventions and strategies to improve the quality of neonatal care.

## Supplementary Material

ihad034_Supplemental_Files

## Data Availability

All raw data generated or analysed during the current study are available from the corresponding author upon request.
