# Supplementary material for: Prevalence of neonatal near miss in Africa: a systematic review and meta-analysis
Source: Int Health. 2023 May 10;15(5):480–9. doi: 10.1093/inthealth/ihad034 (PMC10472883; doi:10.1093/inthealth/ihad034)
Supplement: ihad034_Supplemental_Files [file ihad034_supplemental_files.zip › Additional file 2.docx]

***Additional file 2: Studies search strategies and entry terms from different electronic databases on the prevalence of neonatal near misses in Africa***

**Sample search string for Medline database, EBSCO host Interface**

| **#** | **Query** | **Limiters/Expanders** | **Last Run Via** | **Results** |
| --- | --- | --- | --- | --- |
| S4 | (Africa) AND (S1 AND S2 AND S3) | Search modes - Find all my search terms | Interface - EBSCOhost Research Databases  Search Screen - Advanced Search  Database - MEDLINE | 23 |
| S3 | Africa | Search modes - Find all my search terms | Interface - EBSCOhost Research Databases  Search Screen - Advanced Search  Database - MEDLINE | 612 |
| S2 | Neonate OR Newborn OR Infant OR Near miss Morbidity OR Mortality | Search modes - Find all my search terms | Interface - EBSCOhost Research Databases  Search Screen - Advanced Search  Database - MEDLINE | 101 |
| S1 | Prevalence OR magnitude | Search modes - Find all my search terms | Interface - EBSCOhost Research Databases  Search Screen - Advanced Search  Database - MEDLINE | 505 |

| **Google scholar database** | |
| --- | --- |
| Africa, Neonate OR "Newborn, , prevalence" OR "magnitude" AND " Near miss " OR " Morbidity | 38 |
| **Hinari database** | |
| ((TitleCombined:("neonatal, newborn")) OR (TitleCombined:("near miss, morbidity")) OR (TitleCombined:("Prevalence of near miss")) OR (TitleCombined:("morbidity"))) AND (TitleCombined:(Africa)) | 42 |
