# Supplementary material for: Prevalence of neonatal near miss in Africa: a systematic review and meta-analysis
Source: Int Health. 2023 May 10;15(5):480–9. doi: 10.1093/inthealth/ihad034 (PMC10472883; doi:10.1093/inthealth/ihad034)
Supplement: ihad034_Supplemental_Files [file ihad034_supplemental_files.zip › Additional file 3.docx]

**Appraisal**

Additional file 3: Critical appraisal check list of quantitative studies of neonatal near miss in Africa (Y=yes, N=no/not mentioned); total score=8

| Studies | Q1 | Q2 | Q3 | Q4 | Q5 | Q6 | Q7 | Q8 | Total score |
| --- | --- | --- | --- | --- | --- | --- | --- | --- | --- |
| Tekelab et al. | Y | Y | Y | Y | Y | Y | Y | Y | 8/8 |
| Gebrehana Belay H. et al. | Y | Y | Y | Y | Y | Y | Y | Y | 8/8 |
| Habtamu Abie T. et al. | Y | Y | Y | Y | Y | N | Y | Y | 7/8 |
| C. Ronsmans et al | Y | Y | Y | Y | N | U | Y | Y | 6/8 |
| C. Ronsmans et al | Y | Y | Y | Y | N | U | Y | Y | 6/8 |
| C. Ronsmans et al | Y | Y | Y | Y | N | U | Y | Y | 6/8 |
| Bakari et al. | Y | Y | Y | Y | Y | Y | Y | Y | 8/8 |
| Nakimuli et al. | Y | Y | Y | Y | N | Y | Y | Y | 7/8 |

**Notes:**

Q1 - Were the criteria for inclusion in the sample clearly defined?

Q2 - Were the study subjects and the setting described in detail?

Q3 - Was the exposure measured in a valid and reliable way?

Q4 - Were objective, standard criteria used for measurement of the condition?

Q5 - Were confounding factors identified?

Q6 - Were strategies to deal with confounding factors stated?

Q7 - Were the outcomes measured in a valid and reliable way?

Q8 - Was appropriate statistical analysis used?

**Abbreviations:** Y, yes; N, no; U, unclear.
